# Infective Endocarditis-Associated Pauci-Immune Glomerulonephritis in a Patient With Cryoglobulinemia

**DOI:** 10.7759/cureus.27560

**Published:** 2022-08-01

**Authors:** Stephanie S Pavlovich, William C Bennett, George Terinte-Balcan, Gerald Hladik, Koyal Jain

**Affiliations:** 1 Division of Hospital Medicine, Department of Medicine, University of North Carolina Health, Chapel Hill, USA; 2 Division of Nephrology and Hypertension, Department of Medicine, University of North Carolina School of Medicine, Chapel Hill, USA; 3 Division of Nephropathology, Department of Pathology, University of North Carolina Health, Chapel Hill, USA; 4 Division of Nephrology and Hypertension, Department of Medicine, University of North Carolina Health, Chapel Hill, USA

**Keywords:** antineutrophil cytoplasmic antibody (anca)-associated vasculitis (aav), infection-related glomerulonephritis, pauci-immune glomerulonephritis (gn), cryoglobulinemia, infective endocarditis

## Abstract

Small-vessel vasculitis has a broad differential with similar clinical presentation and laboratory abnormalities, including petechial rashes, neurologic symptoms, glomerulonephritis, and abnormal inflammatory markers. Biopsy-based diagnosis is critical as the treatment varies by etiology. We report a case of a 41-year-old man with diagnosed cryoglobulinemia and hepatitis C presenting with a petechial rash, altered mental status, and acute kidney injury and ultimately found to have proteinase 3 (PR3)-antineutrophil cytoplasmic antibody (ANCA)-positive vasculitis secondary to infective endocarditis. Skin biopsy was consistent with resolving, but nonspecific vasculitis and MRI showed foci of hemosiderin deposition concerning vasculitic lesions. Blood cultures grew *Enterococcus faecalis*, and he was treated with IV antibiotics. Kidney biopsy showed pauci-immune necrotizing focal segmental glomerulonephritis (GN) and diffuse acute tubular necrosis (ATN). After blood cultures cleared, he was initially treated with mycophenolate for worsening renal function. When the patient stopped antibiotics unexpectedly, his kidney function worsened and improved only after immunosuppression was stopped and antibiotics were restarted. This case highlights the importance of renal biopsy in patients with multiple potential etiologies of GN. The case resolution also reinforces that patients with infective endocarditis causing ANCA-associated GN should be treated with antibiotics in addition to, and possibly instead of, immunosuppression.

## Introduction

Small-vessel vasculitis (SVV) broadly comprised two groups of pathology: immune complex-associated disease, including IgA-associated and cryoglobulinemic vasculitis, and pauci-immune disease that is typically accompanied by circulating serum antineutrophil cytoplasmic antibody (ANCA) [1]. Diagnosis can be challenging as many presenting symptoms are shared among different etiologies of SVV, including fever, arthralgias, cutaneous lesions, glomerulonephritis (GN), and neurologic changes. Additionally, ANCA positivity can be associated with infections, including HIV, hepatitis C, tuberculosis, infective endocarditis (in up to 18%-33% of patients), and bartonellosis [2-4], which can present with similar symptoms in the absence of ANCA positivity. Accurate diagnosis is key since the treatment for SVV is dependent on the underlying cause, and in the case of SVV due to infection, immunosuppression may worsen the disease, though this remains unclear.

We present a case of a 41-year-old man with a history of hepatitis C and intravenous drug use (IVDU) with known type III cryoglobulinemia who was ultimately found to have ANCA-associated GN caused by infective endocarditis. Despite treatment of hepatitis C, cryoglobulinemia, and immunosuppression, a full course of antibiotics was required for the improvement of kidney function. This article was previously presented as a poster at the 2021 ASN Kidney Week Meeting on November 4-7, 2021.

## Case presentation

A 41-year-old man with a history of hepatitis C and intravenous drug use presented to our institution with subacute altered mental status, migratory joint pain, and receptive aphasia. One year prior to this presentation, he was diagnosed with hepatitis C, though treatment was initially deferred for unknown reasons. A few months prior to admission, he was noted to have a progressive decline in mental status. He had increasing difficulty with navigation and fluent conversation. One month prior to admission, he presented with a new petechial rash over his legs, aphasia, a new systolic murmur, and a 25-pound weight loss.

His lab work at the time was notable for an elevated rheumatoid factor (14.2 IU/mL), erythrocyte sedimentation rate (ESR: 81 mm/hr), and C-reactive protein (CRP: 32 mg/L). His antinuclear antibody (ANA) was negative, and complement levels were normal (total complement: 46 U/mL). HIV antigen/antibody testing was negative. Serum studies were positive for type III cryoglobulinemia, and he was started on glecaprevir/pibrentasvir for hepatitis C virus (HCV) treatment.

**Figure 1:**
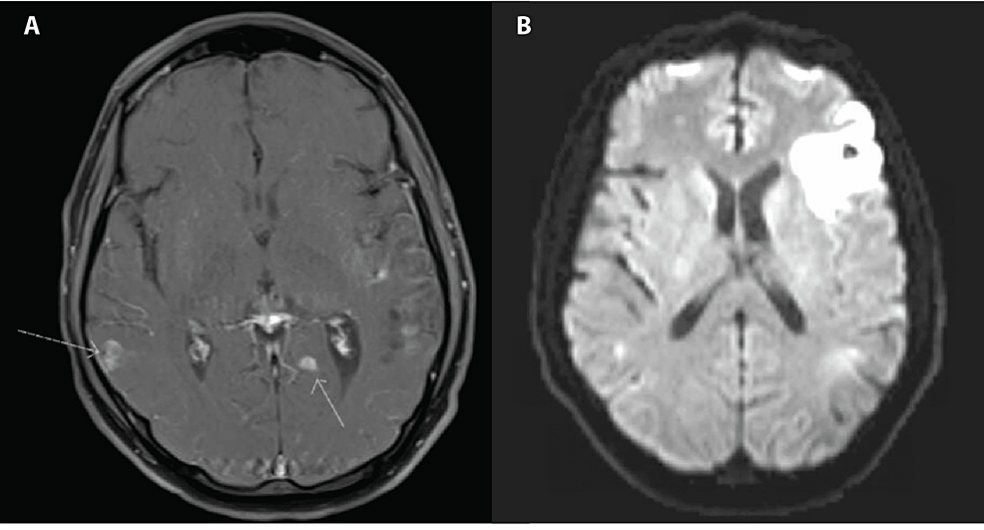
MRI and MRA findings of diffuse focal lesions and infarction A. Nodular foci of enhancement on MRI on T1-weighted image in the axial plane indicated by arrows concerning metastasis, infection, or vasculitis lesions. B. New area of infarct in the left frontal region on MRA on T2-weighted image in the axial plane. MRA: magnetic resonance angiogram.

Two days prior to admission, his aphasia was notably worse. An MRI was performed and showed multiple foci of hemosiderin deposition concerning vasculitis or hemorrhagic metastases (Figures 1A, 1B). Given concern for sequelae of cryoglobulinemic vasculitis, he was brought to the hospital to expedite the workup.

**Figure 2:**
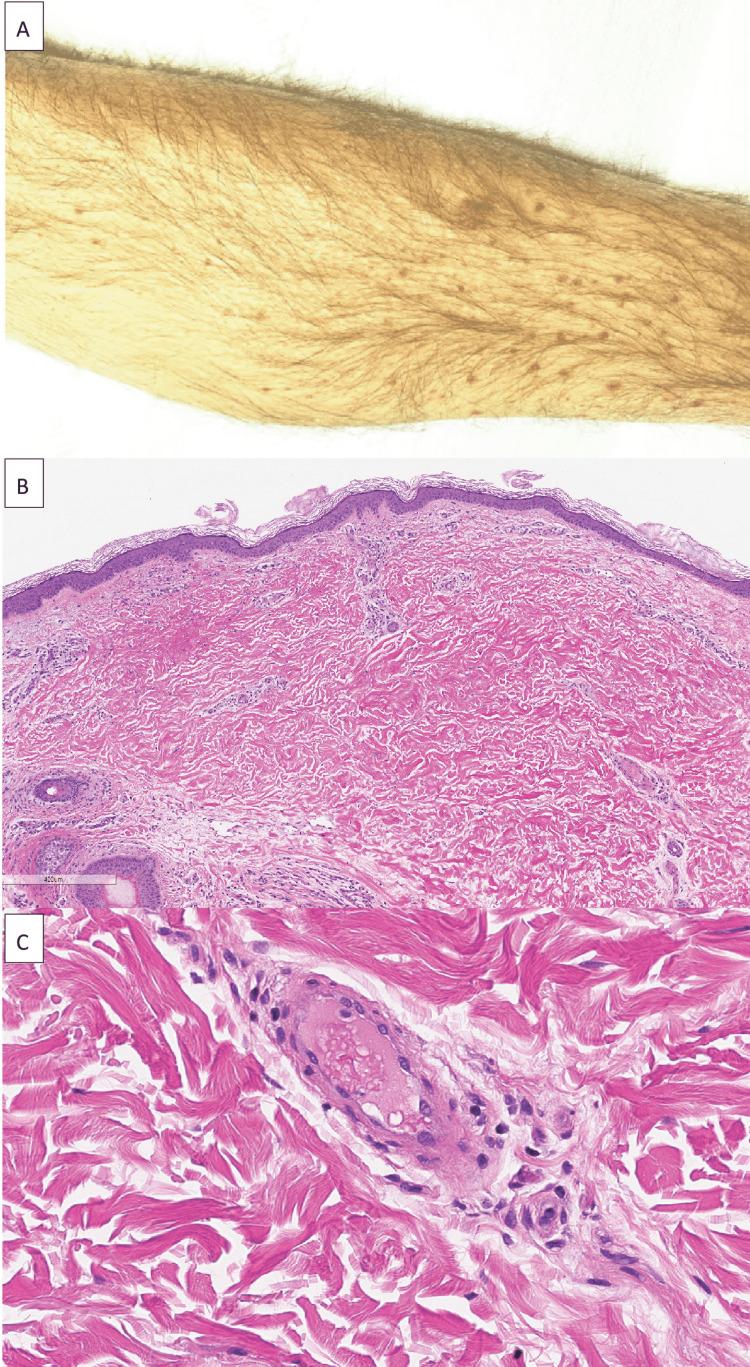
Skin findings on exam and histology A. Petechial rash on lower extremities. B. Skin biopsy showing sparse perivascular inflammation and extravasated erythrocytes with dermal hemorrhage (60×). C. Blood vessel with vascular congestion (300×).

On physical exam on admission, he was initially afebrile but then developed a fever of 38.4°C a few hours after the arrival. His blood pressure was 120/79 mmHg, his heart rate was 66 beats/minute, and his respiratory rate was 16 breaths/minute. His oxygen saturation was 99% on room air. He was non-toxic appearing and had a petechial rash over his shins that coalesced into palpable purpura (Figures 2A-2C). A harsh III/VI murmur was audible loudest at the apex. His liver was palpable 5 cm below the costal margin. His neurologic exam was significant for disorientation to time, receptive aphasia, inability to follow multistep instructions, and bilateral two-beat horizontal nystagmus.

His laboratory studies were notable for WBC of 9.8 x10^9^/L, blood urea nitrogen (BUN) of 15 mg/dL, creatinine of 1.23 mg/dL, CRP of 45.1 mg/L, and rheumatoid factor of 15.9 IU/mL (mildly elevated). Urinalysis (UA) showed no protein, large amount of blood, 13 RBCs, and three WBCs. Urine sediment showed erythrocyte casts and dysmorphic hematuria consistent with glomerulonephritis. C3 was normal at 127 mg/dL, and C4 was normal at 19.4 mg/dL. Cryoimmunofixation showed type III cryoglobulinemia. Cytoplasmic ANCA (c-ANCA) was positive with positive proteinase 3 (PR3) at 49.6 U/mL. A magnetic resonance angiogram (MRA) of the head and neck was done and demonstrated nodular enhancement and hemorrhage bilaterally, evidence of new infarcts in the left frontal lobe, left cerebellum, parietal cortex, and right periventricular white matter (Figure 1B). Vessel walls showed no evidence of vasculitis.

Blood cultures grew *Enterococcus faecalis*, and given his fever and neurological symptoms, a lumbar puncture was performed (WBC: 94, 85% neutrophils, protein: 100 mg/dL, glucose: 44 mg/dL), and cerebrospinal fluid culture grew *Streptococcus sanguinis*. A transthoracic echocardiogram showed moderate mitral regurgitation and evidence of mitral valve vegetation. A transesophageal echocardiogram confirmed the presence of a 1.2 cm mobile vegetation on the anterior leaflet of the mitral valve. The patient was started on intravenous ampicillin 2 g every six hours (renal dosed) and ceftriaxone 2 g every 12 hours to treat his endocarditis and potential meningoencephalitis.

**Figure 3:**
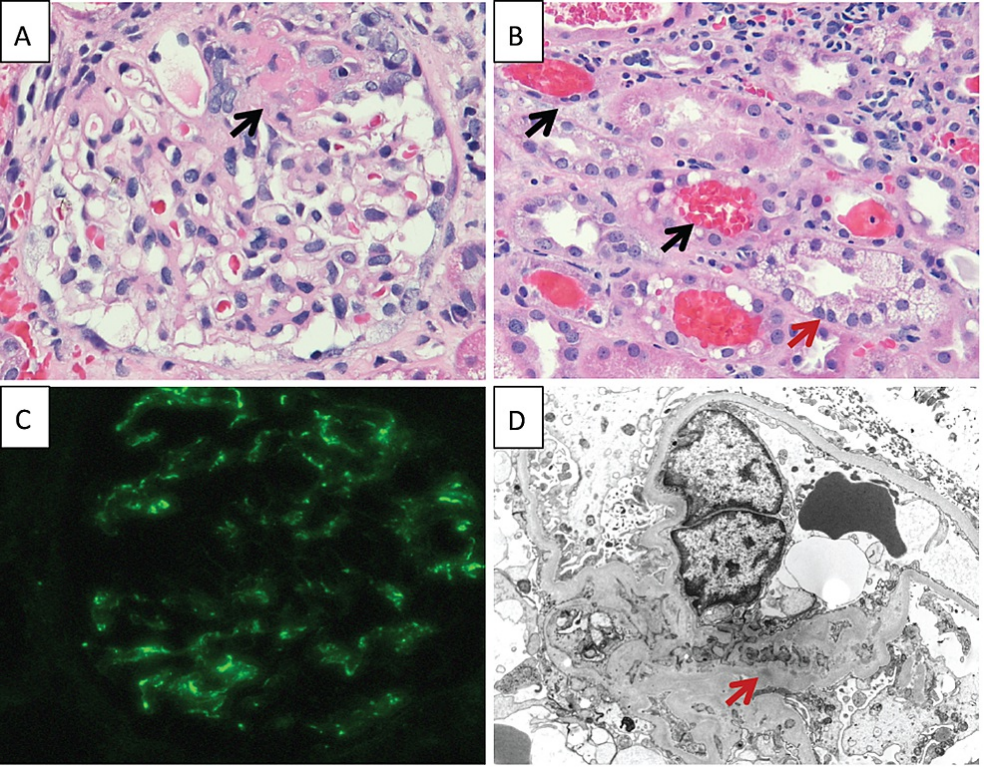
Kidney biopsy pathology A. Glomerulus with a small segment of fibrinoid tuft necrosis (black arrow) on light microscopy (HE stain, 200×). B. Several red blood cell casts (black arrows) located in tubular lumens and a tubule with marked cytoplasmic vacuolation of the tubular epithelial cells (red arrow) (HE, 200×). C. Mild (1+) granular staining for C3 in the mesangial areas of the glomerulus on immunofluorescence (200×). D. Electron microscopy image showing a mesangial region with very small, rare electron-dense deposits (red arrow). HE: hematoxylin and eosin stain.

Due to ongoing arthralgias felt to be secondary to SVV, he was started on hydroxychloroquine as a trial and to avoid steroid use with active bacteremia. His rash was evaluated by dermatology, and the skin biopsy showed nonspecific inflammation with resolving vasculitis (Figure 2). Due to worsening acute kidney injury (AKI) with dysmorphic hematuria on urine sediment, the patient underwent a kidney biopsy, which showed pauci-immune necrotizing focal/segmental GN in 5%-10% of glomeruli as well as diffuse acute tubular necrosis (ATN) (Figures 3A-3D). As the blood cultures had cleared and the patient had an AKI (peak creatinine: 2.66 mg/dL) secondary to pauci-immune glomerulonephritis, he was started on mycophenolate.

He was discharged on a six-week course of IV antibiotics (ampicillin 2 g every six hours renally dosed and ceftriaxone 2 g every 12 hours via a peripherally inserted central catheter) from the day of clearing his cultures. His creatinine on discharge was stably around 2.4 mg/dL, which downtrended on postdischarge follow-up to 1.6 mg/dL. Unfortunately, due to logistical difficulties, his antibiotics were stopped early. On follow-up one month after discharge, his urine sediment showed hematuria, RBC casts, degenerative granular casts, and acanthocytes. Creatinine again rose to a peak of 1.87 mg/dL, and repeat blood cultures were positive for *E. faecalis*. Mycophenolate was stopped, and he was restarted on antibiotics (IV ampicillin 2 g every four hours and ceftriaxone 2 g every 12 hours). His central catheter was replaced, and he was treated for a six-week course via a home infusion pump. His renal function improved gradually with creatinine on the most recent follow-up of 1.40 mg/dL and bland urine sediment.

His mental status and aphasia improved gradually with speech therapy and completion of antibiotics. He was followed by infectious disease consultants, and given the uncertain clinical significance of *S. sanguinis* on spinal fluid culture as well as the patient’s symptom improvement on appropriate antibiotic coverage, lumbar puncture was not repeated. After clinical improvement and completion of antibiotics, he did ultimately undergo bioprosthetic mitral valve replacement; valve cultures done at the time of cardiac surgery were negative. Bioprosthetic valve function on subsequent transthoracic echocardiogram was normal.

## Discussion

SVV has a broad differential diagnosis that can be difficult to narrow based on clinical manifestations and laboratory findings alone. We present a patient with known type III cryoglobulinemia and suspected cryoglobulinemic vasculitis but ultimately found to have PR3-ANCA-associated vasculitis caused by *E. faecalis* endocarditis. This case has several important teaching points. First, a kidney biopsy is critical in patients with multiple potential etiologies of GN. Based on history and accumulated laboratory data on initial presentation, this patient was strongly suspected to have cryoglobulinemic vasculitis. When he subsequently developed an AKI in the presence of multiple systemic manifestations of mixed cryoglobulinemia while on antiviral therapy, the next step in treatment presented a clinical challenge. The mainstay of treatment for hepatitis C-associated cryoglobulinemia is the use of direct antiviral acting agents to target a virologic response [5,6]. Beyond treating the underlying cause, immunosuppressive agents such as glucocorticoids, cyclophosphamide, or rituximab can be used for severe cryoglobulinemic vasculitis. Given the presence of bloodstream infection and ANCA positivity, we pursued a kidney biopsy for diagnosis, which did not show a membranoproliferative GN (as would be expected with cryoglobulinemic kidney injury) and was instead consistent with ANCA-associated kidney injury.

The second teaching point is that infective endocarditis can mimic systemic vasculitis [3,7,8]. Patients with a history of IVDU in particular can have multiple potential etiologies of systemic lesions with a vascular distribution given the concern for HIV, hepatitis B or C, drug-induced vasculitis, and bloodstream infection. With regard to endocarditis, up to 18%-33% of patients can present with ANCA positivity, typically PR3-ANCA, but potentially myeloperoxidase (MPO)-ANCA or dual positivity as well [3,4].

Finally, this case highlights the importance of antibiotic therapy in PR3-ANCA vasculitis secondary to infective endocarditis. Given the circumstances of our patient’s incarceration, antibiotics were stopped and the effect of recurrent bacteremia on kidney function was clear. On the patient’s second admission, we elected to hold immunosuppression and treat with antibiotics, which ultimately led to improvement of his renal function. It is controversial whether infection-associated vasculitis should be treated with immunosuppression or not. As ANCA-positive infective endocarditis is relatively rare, it is difficult to compare outcomes from antibiotic therapy alone or antibiotics along with immunosuppression. Zhang et al. report a series of eight patients who were treated with both immunosuppression and antibiotics and recovered well from ANCA-positive infective endocarditis [3]. However, there are also other reports of patients who may have had delayed antibiotics due to an initial diagnosis of ANCA vasculitis; in a series of patients with chronic infection-associated vasculitis, only two of 11 patients were treated with immunosuppressive therapy in addition to antibiotics and both died [9,10]. Our patient’s case illustrates that antibiotics are certainly necessary, though in cases of severe vasculitis, they may not be sufficient for recovery [8].

## Conclusions

SVV is easily confused for infectious processes, particularly infective endocarditis given its multiorgan system disease manifestations and the presence of several serologic markers in both conditions. If there is any diagnostic uncertainty, obtaining a tissue diagnosis is key. This is especially true for renal disease, as inappropriate or delayed treatment can lead to rapid loss of residual kidney function. If an infectious cause is an underlying pathology, antibiotics are a crucial part of management. There remains debate regarding the utility of immunosuppression in these patients, but antibiotics should not be delayed.
